# Neural Stem Cells as Potential Glioblastoma Cells of Origin

**DOI:** 10.3390/life13040905

**Published:** 2023-03-29

**Authors:** Alba Loras, Luis G. Gonzalez-Bonet, Julia L. Gutierrez-Arroyo, Conrado Martinez-Cadenas, Maria Angeles Marques-Torrejon

**Affiliations:** 1 Department of Medicine, University of Valencia, 46010 Valencia, Spain; 2 Department of Medicine, Jaume I University of Castellon, 12071 Castellon de la Plana, Spain; 3 Department of Neurosurgery, Castellon General University Hospital, 12004 Castellon de la Plana, Spain

**Keywords:** glioblastoma, neural stem cells, cell of origin

## Abstract

Glioblastoma multiforme (GBM) is the most malignant brain tumor in adults and it remains incurable. These tumors are very heterogeneous, resistant to cytotoxic therapies, and they show high rates of invasiveness. Therefore, patients face poor prognosis, and the survival rates remain very low. Previous research states that GBM contains a cell population with stem cell characteristics called glioma stem cells (GSCs). These cells are able to self-renew and regenerate the tumor and, therefore, they are partly responsible for the observed resistance to therapies and tumor recurrence. Recent data indicate that neural stem cells (NSCs) in the subventricular zone (SVZ) are the cells of origin of GBM, that is, the cell type acquiring the initial tumorigenic mutation. The involvement of SVZ-NSCs is also associated with GBM progression and recurrence. Identifying the cellular origin of GBM is important for the development of early detection techniques and the discovery of early disease markers. In this review, we analyze the SVZ-NSC population as a potential GBM cell of origin, and its potential role for GBM therapies.

## 1. Introduction

### 1.1. Glioblastoma

Glioblastoma multiforme (GBM) is the most complex, aggressive, and deadly human brain tumor. The World Health Organization classifies GBM as a Grade IV tumor [1]. Today, despite surgery, chemotherapy, and radiotherapy treatments, the survival rate for these tumors is very low and it oscillates around 16 months [2,3]. There are no treatments that have significantly prolonged survival, with 5-year survival rates of about 6% [4]. Much of the failure of conventional therapies is due to the existence of inter- and intra- tumor heterogeneity [5,6,7].

Based on the cancer genome atlas (TCGA), GBM has been classified into four subtypes: proneural, neural, classical, and mesenchymal, with the mesenchymal subtype being the most aggressive subtype with the worst prognosis [6,8,9,10]. Later studies propose a different classification system based on IDH (Isocitrate dehydrogenase) gene mutation status: tumors classified as IDH wild-type, IDH mutant, and patients whose full IDH analysis cannot be determined called GBM NOS (not otherwise specified) [1]. Another reason for intra-tumoral heterogeneity is the presence of cells with stem-like features, called glioma stem cells (GSCs). These cells can self-renew, differentiate into different lineages, show high resistance to cytotoxic and radio- therapies, and display high tumorigenicity [9,11,12,13].

Age-adjusted annual incidence values of GBM fluctuate between 0.6 and 3.7 per 100,000 people [14]. In general, GBM is diagnosed in elderly patients, with a median age of about 64 years. Incidence rates increase with age, peaking between 75 and 84 years [15]. Primary GBM is usually diagnosed between 55 and 64 years of age, while secondary GBM is diagnosed around 40 years of age [16]. However, GBM is extremely rare in children [15].

The most evident sex-related difference in GBM is the prevalence rates. Malignant brain tumors are more frequent in males than females, with GBM rates 1.6 times higher in men than in women. However, there is no difference in the incidence of low-grade gliomas. Regarding GBM subtypes, primary tumors are more frequent in males, but secondary tumors appear more frequently in females. Both in human and animal research, higher survival is found in females [4,17,18,19,20,21]. Unfortunately, tumor recurrence is unavoidable and appears in a short time period, irrespective of gender.

Most GBMs occur as primary tumors (90%), without the existence of a previous tumor, and are frequent in elderly patients. On the other hand, in younger patients, GBMs usually appear following a low-grade lesion, and therefore, they occur as secondary tumors. Both tumors do not differ histologically, but secondary tumors are genetically characterized by the presence of a mutation in the *IDH1* gene. Primary and secondary GBMs are therefore called the *IDH1* wild-type and *IDH1* mutant, respectively [1,22]. Both GBM subtypes usually present mutations in the deregulation of different pathways, such as the tumor-suppressor p53 signaling pathway (*CDKN2A*, *MDM2*, *TP53* genes), *NF1* (neurofibromin 1) gene mutations, or *TERT* (telomerase reverse transcriptase) gene mutations [10,23,24]. Likewise, primary GBMs are characterized by *EGFR* overexpression, *PTEN* mutations, and *CDKN2A* (p16) deletions, while the most common and detectable alteration in secondary GBM, other than *IDH1* gene mutations, is the presence of *TP53* gene mutations [6]. 

Six subgroups of DNA methylation, DNA copy number alterations, and transcriptomic patterns have been found in adult and pediatric GBMs. Two of these subgroups have been found to affect essential amino acids (K27 and G34) in histone H3.3 and are predominantly found in children and adolescent patients [25].

### 1.2. The Potential GBM Cell of Origin

The cellular origin of GBM refers to the ordinary cell that is susceptible to oncogenic mutations and therefore, has the physiological potential to transform into a cancerous cell and generate a tumor. Identifying the cellular origin may therefore lead to the development of more effective and perhaps preventive therapies. The definition of the cell of origin, as the cell type initiating a glioma, is therefore different from that of the glioma stem cell (GSC), since the latter concept refers to a cellular subtype existing in the tumor that is acting as a reservoir for tumor regeneration.

**Figure 1:**
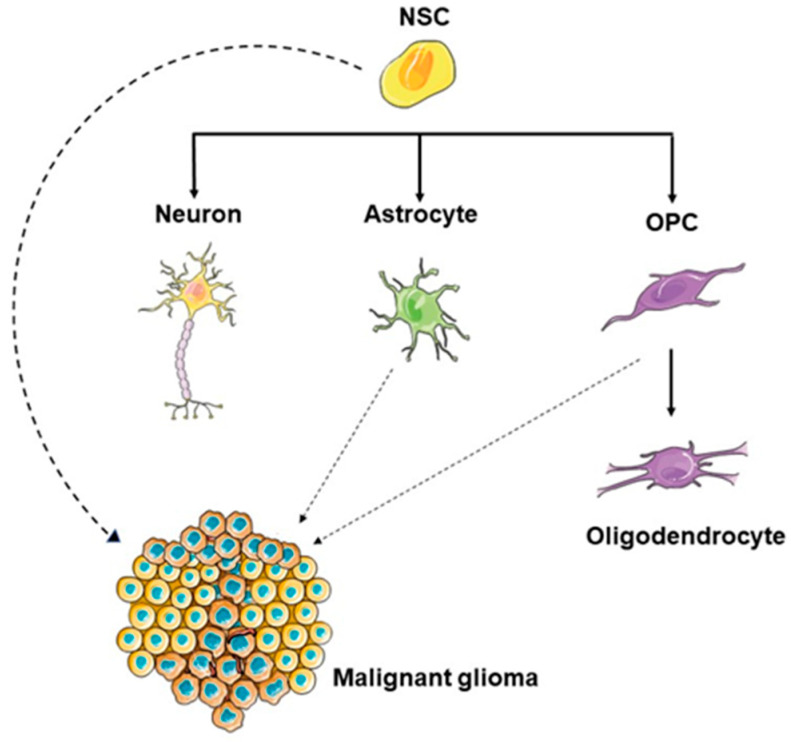
The glioma cell of origin. NSCs are undifferentiated cells with self-renewal and multipotent capacities. They give rise to neurons, astrocytes, and OPCs. NSCs can be mutated and converted into glioma stem cells (GSCs).

To determine the cellular origin of gliomas, it is essential to understand the development of glial cells in mammals [26]. During the development of the central nervous system (CNS), NSCs reside in the ventricles of the brain. However, in the adult brain, they will only remain in two specific regions, the subventricular zone (SVZ) and the subgranular zone (SGZ) of the dentate gyrus in the hippocampus [27,28]. These NSCs will generate glial cells (astrocytes and oligodendrocytes) and neurons [27,29,30,31]. At the end of embryonic development, most progenitor cells undergo terminal differentiation, with the exception of oligodendrocyte precursor cells (OPCs). These OPCs remain undifferentiated and can proliferate in the adult brain [32,33]. In addition to NSCs and OPCs, astrocytes, which are differentiated cells, retain some proliferative capacity, particularly after brain injury [34]. Because NSCs, OPCs, and astrocytes all have some regenerative potential, they all present a potential source of cells for initiating gliomas [35,36] (Figure 1).

In order to become tumorigenic, astrocytes require a multistep process. Firstly, they must dedifferentiate and, secondly, they need to acquire tumor characteristics, including oncogene and tumor-suppressor modifications. In comparison, NSCs and OPCs are already proliferative and therefore only require oncogene and/or tumor-suppressor alterations. Thus, they present a more feasible GBM cell of origin. A recent publication shows that astrocyte precursors and OPCs can generate brain tumors involving driver mutations in NSCs that promote carcinogenesis after committing to the oligodendrocyte line [37]. Clinically, it has been shown in humans that NSCs, after transplantation as potential therapies for neurodegenerative diseases, can participate in the process of brain tumor formation [38].

### 1.3. The Human SVZ 

**Figure 2:**
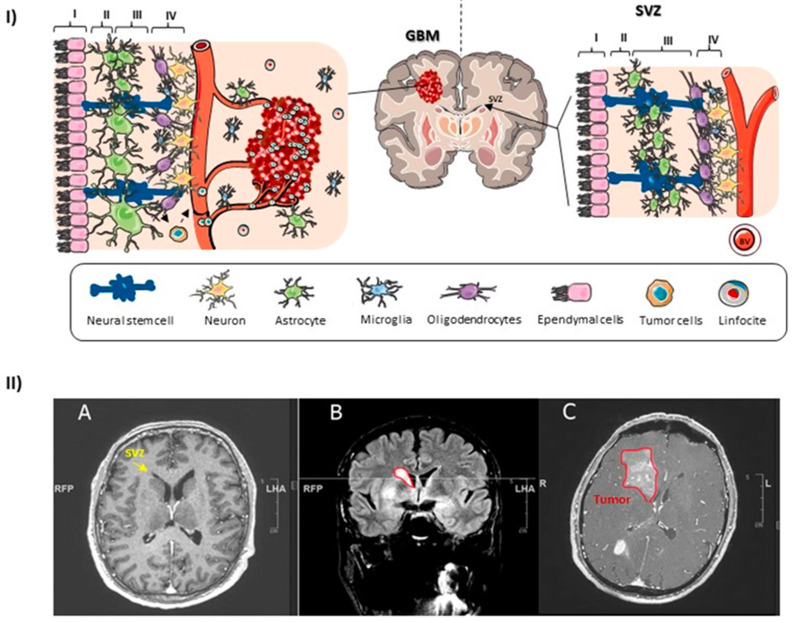
The adult human SVZ. (**I**) Illustration of the adult human SVZ and adjacent GBM. Left panel shows a drawing with the four layers of the human SVZ (I–IV) and an adjacent GBM close to this area. Ependymal layer (layer I) is lining the lateral ventricle. NSCs are represented in navy blue. These cells are in contact with the CSF and blood vessels (BV). NSCs could acquire driver mutations, become GSCs, and generate the tumor mass (red mass). (**II**) A patient was diagnosed with *IDH1* wild-type GBM by biopsy and is showing an early contrast-enhanced axial T1-weighted MRI with non-enhancing areas (**A**), and a small hyperintensity in FLAIR sequence imaging located in the lateral wall of the right lateral ventricle (**B**). After a month, we can observe progression of the tumor, with an increase in T1 contrast enhancement (**C**).

The SVZ is one of the adult mammalian neurogenesis niches [39,40,41]. It is well known that NSCs have astrocytic features and self-renewal and multipotency capacities (differentiation into neurons, oligodendrocytes, and astrocytes) [27,39,42]. The SVZ area lines the lateral ventricles of the brain, and it is composed of different types of cells in a well-organized pattern (Figure 2) [43]. Although neurogenesis in this area persists throughout life, from fetal to adult stages, this neurogenic capacity undergoes a significant decrease with age [41,44]. Compared to the rodent SVZ, the human SVZ is organized differently. It is divided into four layers: (i) the ependymal layer, which lines the lateral ventricle where CSF (cerebrospinal fluid) flows and is composed of multi-ciliated ependymal cells; (ii) the hypocellular layer, which contains extensions of astrocytes; (iii) a layer containing three distinct types of astrocytes; and finally, (iv) a layer with myelinated axons and oligodendrocytes [41]. In the human SVZ, in contrast to the rodent SVZ, there is no evidence of fast proliferating precursor cells, and neither the existence of a rostral migratory stream (RMS) with neuroblasts migrating to the olfactory bulb (OB) [39,45,46]. In the pediatric human SVZ, NSCs generate neurons that migrate both to the prefrontal cortex and to the forebrain [47,48]. In contrast, neurogenesis is a rare event in the adult human SVZ, although there is restricted neurogenesis in the nearby striatum [39,49].

### 1.4. Similarities between NSCs and Glioma Stem Cells

Many characteristics are shared between NSCs and GSCs, as seen by genome-wide CRISPR-Cas9 screens [50]. SOX2 is one of the most important players for the maintenance of both NSCs and GSCs, and SOCS3, a protein responsible for maintaining stemness in NSCs, is also an important GBM-specific fitness gene in GSCs [50]. There is also evidence for developmental patterns being activated in GBM. For example, some genes such as *CDK6*, *SQLE*, and *DOTIL* have great scores with NSCs. However, *JUN* and *SOX9* display lower scores [50]. These findings indicate that gene networks shared with NSCs are important for GSC maintenance, while GBM-specific genes promote the generation of tumorigenic GSCs [50].

Proteomic analyses have also revealed similarities between NSCs and GSCs [51,52,53,54,55,56,57,58]. Multiple signaling pathways that are necessary for self-renewal have been shown to be active in both NSCs and GSCs, and vimentin is overexpressed in both cell types [52,53,54,55,56,57,58,59]. It is known that the Notch pathway is required to maintain the undifferentiated state of NSCs by inhibiting proneural gene expression. This pathway has been found to be upregulated in GSCs [60,61]. The BMP pathway promotes gliogenesis and inhibits neurogenesis in NSCs [62,63]. BMP has been shown to promote astrocyte differentiation and decrease proliferation in NSCs and GSCs [64,65,66,67]. The Wnt and β-catenin signaling pathways control both NSC and GSC proliferation [68,69]. In addition, the sonic hedgehog pathway regulates the self-renewal of both NSCs and GSCs through Gli1 [56,70]. STAT3 is necessary for proliferation and multipotency regulation in NSCs and GSCs [71,72]. Some growth factors that are required during development are also secreted by GSCs, such as the endothelial growth factor (VEGF), the basic fibroblast growth factor (bFGF), the transforming growth factor alpha (TGF-alpha), and the stromal-derived factor (SDF1) [73,74,75,76,77,78]. One study examined 19 chromosome proteins in GSCs and encountered the upregulation of many molecular pathways associated with development [79]. Together, these molecular similarities between NSCs and GSCs, involving stemness and development signatures, indicate NSCs as the potential GBM cell of origin.

### 1.5. NSCs of the SVZ as the GBM Cell of Origin

Evidence suggests that GSCs could originate from SVZ-NSCs, as these cells are more susceptible to oncogenic transformation than SGZ-NSCs of the hippocampus or differentiated cells [80]. Furthermore, human SVZ-NSCs are more susceptible to malignant transformation as they mainly generate OPCs [81]. The SVZ is chemo-attractive for glioma cells, and microglia are supportive cells for neurogenesis [82]. SVZ-NSCs line the lateral ventricles and are in contact with the CSF, which are rich in factors that could promote and maintain the tumor [83,84]. SVZ-NSCs do not display an expression of *HOPX*, a tumor-suppressor gene, and mutations in the *TERT* gene promoter could activate senescence [80,85].

The regenerative plasticity and the developmental potential of NSCs, with their intrinsic properties—self-renewal, proliferation, apoptosis, and senescence avoidance—make them a suitable potential glioma cell of origin [86]. Clinically, both a complex cellular composition and the existence of multilineage markers within the tumor have been observed, suggesting ongoing differentiation. However, there may also be dedifferentiation of the committed cell progenitors. Cultured cancer cells tend to exhibit numerous NSC markers, develop renewable tumor spheres, and differentiate into numerous cell types upon serum stimulation, all of which, are NSC features [87]. 

Although these data are promising, it remains difficult to definitively identify the GBM cell of origin using endpoint studies. Confirmation must come from studies of genetically modified animal models, in which physiologically significant tumor-driver mutations allow for the analysis of early events in tumor formation [88,89].

In order to precisely identify the cellular origin of the tumor in vivo, it is critical to use specific methods to produce mutations only in selected cells. For example, retrovirus vectors are widely used to induce mutations in a small number of cells or in restricted areas of the brain [90]. This makes it possible to discern between tumors arising from resident cells versus cells that break away from the tumor mass to colonize distant areas. With the retroviral approach and using different combinations of *PTEN*, *TP53*, *NF1*, and *RB1* mutations, NSCs have been identified as a possible cellular origin of GBM in mouse models [91,92,93]. In addition, tumor formation was found to be dependent on the niche in which the cells were located. This type of study tests the ability of tumors to originate in specific regions of the brain and also shows that SVZ-NSCs are more readily converted into cancerous cells than differentiated cells outside this region [93]. These studies show the tendency of NSCs to serve as the GBM cell of origin; however, they do not discount the fact that alternative cell types, especially progenitor cells, could also be putative cells of origin for GBM.

Recently, research has shown the first genetic confirmation of the cell of origin in human GBM [37]. This study shows that in 56.3% of cases of *IDH1* wild-type GBM, the SVZ tissue contained driver mutations in genes such as *TP53*, *PTEN*, and *EGFR* [37]. Additionally, 42.3% of patients had *TERT*-promoter mutations in the SVZ tissue as well as in the tumor tissue [37]. Furthermore, they also demonstrated that SVZ-NSCs harboring oncogenic mutations were able to travel and form GBMs in remote brain areas [37]. These findings reveal that human SVZ cells can harbor cancer driver mutations, at least in *IDH1* wild-type GBMs. Remarkably, *TERT*-promoter mutations in the SVZ area were found in all patients with *IDH1* wild-type tumors. SVZ-NSCs exhibit only a partial self-renewal capacity and therefore, they could avoid telomere shortening and increase the possibility of acquiring driver mutations [37]. However, this study did not find evidence of the cell of origin in *IDH1*-mutant GBMs. Moreover, in 7 out of the 16 GBM samples, they did not find mutations in the SVZ, which suggests that SVZ-NSCs may not be the cell of origin for all *IDH1* wild-type GBMs [37]. The concept of dedifferentiation as an alternative for the cell of origin has been established using mouse experimentation, and no confirmation in humans has been shown in this study. They also generated a murine model of *PT53*, *EGFR*, and *PTEN* mutations through genome editing, with the same mutations that were present in the SVZ of GBM patients [37]. Subsequently, 90% of the animals with these mutations ended up developing brain tumors, and 67% of these tumors were found in distant areas from the SVZ [37]. In addition, SVZ NSCs carrying these mutations were able to migrate a great distance to the olfactory bulb (OB) and differentiate into neurons, without the generation of tumors [37]. It is therefore vital to understand the niche signaling in different areas of the brain, since this could promote tumor generation or differentiation. Understanding niche signaling will be of critical importance in the design of new therapies for this disease. 

## 2. Materials and Methods

An exhaustive bibliographic search of English-language publications available in PubMed and Scopus databases was performed. No limits were used on the publication dates of the articles. The keywords used for the bibliographic search in this article were as follows: GBM, cell of origin, and NSCs. In addition, the reference lists of relevant articles and published reviews were revised in order to identify additional studies. Publications in a language other than English were excluded. No studies were excluded a priori due to poor design or data quality. Articles were first selected by titles and abstracts. If the information in the abstract was not detailed, the full text was reviewed.

## 3. Discussion

Recent evidence indicates that the heterogeneity of GBM could be linked to its cell of origin and have stem cell properties [13,94]. For a long time, neuroscientists have focused their attention on the SVZ as the potential brain area of GBM origin (or at least contributor). Scientific evidence shows that more than 50% of the GBMs are located near the SVZ, and that this location is associated with poor prognosis [95,96]. NSCs are close to the lateral ventricles and the recruitment of these cells to the tumor could enhance GBM aggressiveness. It has also been shown that the relationship between GBM and the SVZ is associated with multifocality and recurrence [82,97]. In addition, some studies in humans suggest that the enhanced radiotherapy of the SVZ is associated with a longer survival of GBM patients [2,3]. 

However, there are still many human brain tumors situated in distant areas other than the SVZ [98]. This could be explained by the asymmetric division of NSCs into two daughter cells with different proliferative and migrative features [98,99]. One of these daughter cells could stay in the SVZ, while the other could migrate a great distance to form the tumor mass in a different brain area [100]. These migrating tumor cells are possibly GSCs that have previously been transformed within the SVZ [100]. In fact, a similar event occurs during brain development, where radial glial cells migrate to the cortex and then differentiate [26]. The fact that gliomas usually recur nearby could also be explained, since secondary tumors derive from primary tumors and do not form in the SVZ. Classical therapies (surgery, radiation, and chemotherapies) remove the bulk of the tumor mass, but it is thought that some cells are able to survive; these cells could be the source of the cells that are responsible, in a short period of time, for the formation of secondary tumors that are close to the primary site [1].

There is evidence that secondary GBMs (*IDH1*-mutant) could have different potential cellular origins to primary GBMs (*IDH1* wild-type) [101]. Accordingly, different genetic and animal approaches should be considered in order to understand the mechanisms through which cells acquire mutations and undergo lineage specification during gliomagenesis [102]. In this context, it would be interesting to study how the niche influences GSCs fate and its impact on the development of the different subtypes of GBM.

Another fascinating prospect is that secondary GBMs are derived from the SVZ, where a primary GBM may “replenish” NSCs from the SVZ [103]. This circumstance of attracting regular cells to become part of the tumor mass has been observed through the production and release of trophic molecules into the tumor niche [104]. This may also be contributing to tumor heterogeneity [105,106]. Additionally, in rodent models, it has been shown that NSCs are highly trophic and migrate to the tumor following these signals [105,107,108]. These findings could be important to understand tumor resistance to current therapies, as tumor-free areas may be occupied by migrating cells [98].

Nevertheless, there is evidence that associates GBM with the SVZ. A complex in vitro 3D model or organoid system similar to the SVZ and its niche would be necessary to accurately study this association. Using this model, the structure and cytoarchitecture of the SVZ would be recreated exactly, and different regions of the brain could be studied as potential areas involved in tumor formation. Such studies could greatly enhance the possibilities of finding new potential therapeutic targets.

Although this review has focused on SVZ NSCs as the best candidate for the GBM cell of origin, all three candidate cell types (NSCs, astrocytes, and OPCs) have established regeneration potential in the brain. This implies that the tumorigenic transformation of these cells is determined by their oncogenic mutations and their regeneration capacities. GBM is a very diverse disease and it presents a great diversity of clinical and histopathological characteristics. This could be due to GBMs being intrinsically derived from different cell types.

Clinical data from GBMs may suggest evidence for putative cellular origins; however, drawing conclusions from clinical data has proven complex, as human tumors are usually studied at the terminal stage [82]. An important issue to consider is that the different subtypes of GBM defined by TCGA data could be due to different cellular origins [82]. In fact, gene expression profiles of the proneural GBM subtype are compatible with that of OPCs, and the profiles of other GBM subtypes correspond to those of NSCs or astrocytes. In addition, different developmental potential cells of origin have also been suggested [109] to explain the different potential GBM cell of origin; multiple cell types could function as the developmental origins of different GBM subtypes. For this it would be necessary to dissect each transforming process and analyze them temporally and spatially, frame by frame, in order to obtain the applicable information for therapeutic purposes.

The use of markers to track tumor cells of origin can be difficult, as studies are performed at the later stages of tumor evolution. It is therefore important to carry out studies of cell type-specific gene expression profiles using transcriptomic analysis instead of candidate gene analyses [6,110]. In this way, genomics may produce significant advances in the knowledge of novel molecular mechanisms involved in gliomas. 

Murine genetic models are extremely useful in allowing us to trace the cellular origin of GBM from its initial stages. In this way, it is possible to obtain a temporal and spatial analysis of tumor formation. In addition, these animal models may be used in preclinical trials of potential drugs. However, the differences between human and mouse cell populations must be considered, and the upcoming results must therefore be carefully translated to human cells and tissue before clinical translational approaches. 

The study of the different cell states of NSCs (proliferation, quiescence, and differentiation) also aids our understanding of the possible cell states of the GBM population and potential therapies. Focus is needed on eliminating the proliferating tumor population through antiproliferative therapies such as temozolomide (TMZ), radiotherapy, and EGFR inhibitors. Unfortunately, the potential for cellular reprogramming and dedifferentiation within the tumor makes conventional therapies ineffective. Targeting specific tumor characteristics such as proliferation (chemotherapy, radiotherapy, and EGFR inhibitors) and network integration (e.g., ion channel modulators) can eliminate specific tumor populations and may induce differentiation to improve GBM treatment efficiency. For example, it is known that the disruption of OLIG2 induces cell differentiation [111]. Similarly, the removal of growth factors and the addition of bone morphogenic protein (BMP4) induces the cell cycle exit and differentiation of GSCs [112]. However, this cell differentiation depends on the disruption of the signaling pathway between the transcription factor and the receptor tyrosine kinase’s signaling. Moreover, even if there were targeted therapies for differentiated cells, not all cells differentiate. It has been shown that differentiation is induced by reversible histone modifications of CpG islands [112]. Consequently, as these histone modifications are reversible, cells re-enter the cell cycle. Therefore, cell differentiation as an antitumor therapy must be terminal. Neurogenic differentiation and reduced tumor formation have been shown to be diminished by Notch signaling or MASH1 inhibition in GBM [113]. The most effective antitumor therapy would therefore be one that completely differentiates tumor cells, whether proliferating, dormant, or any cell type, from neuronal differentiation, which is irreversible. The side effect of such therapies on the differentiated population such as neurons and glia would also have to be considered.

The fundamental aim in studying the cell of origin of GBM is to find new therapeutic targets for this disease [114]. Different origins may imply different prognoses and responsiveness to treatments. Understanding the cellular origin would therefore allow us to classify patients and develop more personalized and effective treatments. Drugs could be developed considering different parameters. Firstly, specific membrane proteins would allow for the use of selective drug administration, reducing the toxicity to other brain cells [115]. Secondly, the possibility of susceptibility to certain genetic mutations and therefore to the activation or inhibition of certain signaling pathways could be avoided [18]. It would be effective to target the interactions between signaling pathways and tumorigenic mutations. Finally, knowledge of the cell of origin would further the connection between oncology and the field of regenerative medicine. For example, the study of the enhanced self-renewal capacity in NSC and tumor progenitor cells could shed light on NSCs expansion in regenerative medicine. Similarly, understanding the differentiation process in NSCs and progenitors could be a challenge for GBM therapy; this strategy has great potential because it would reduce tumor progression while avoiding cytotoxic drug resistance.

## 4. Conclusions

In conclusion, studies on the GBM cell of origin have not yet reached a clear conclusion. In fact, it could almost be stated that these studies have just begun. The study of adult neurogenic niches is currently a very active research topic. In addition to their function as neural cell generators, new data have identified neurogenic niches as modulators of brain tumors (as they are partly responsible for both their origin and maintenance, as well as a source of recurrence). Due to its special characteristics, the SVZ seems to be the neurogenic niche that could be responsible for the generation of gliomas. There are different cell types in this region of the brain that could act as the potential cellular origin of GBM tumors.

In order to progress in both the understanding and treatment of this devasting disease, it is necessary to implement innovative technologies and assimilate new concepts into our current knowledge. While our knowledge progresses faster than those GSCs, effective treatment for GBM should be within our scope.

## Data Availability

No new data were created or analyzed in this study. Data sharing is not applicable to this article.
